# Gene alterations as predictors of radiation-induced toxicity in head and neck squamous cell carcinoma

**DOI:** 10.1186/s12967-021-02876-5

**Published:** 2021-05-17

**Authors:** Whitney Sumner, Xenia Ray, Leisa Sutton, Daniel Rebibo, Francesco Marincola, Parag Sanghvi, Vitali Moiseenko, Ida Deichaite

**Affiliations:** 1 Department of Radiation Medicine and Applied Sciences, University of California San Diego, La Jolla, CA USA; 2 Moores Cancer Center, University of California San Diego, La Jolla, CA USA; 3 Science, Refuge Biotechnologies, Menlo Park, CA USA

**Keywords:** Predictive biomarkers of radiation toxicity, Head and neck squamous cell carcinoma, Radiogenomics, BRCA2, ERBB3, RT dosimetric data, TNFAIP3, HNF1A, SPTA1, CASP8

## Abstract

**Background:**

Optimizing the therapeutic ratio for radiation therapy (RT) in head and neck squamous cell carcinoma (HNSCC) is uniquely challenging owing to high rates of early and late toxicity involving nearby organs at risk. These toxicities have a profound impact on treatment compliance and quality of life. Emerging evidence suggests that RT dose alone cannot fully account for the variable severity of RT-related adverse events (rtAEs) observed in HNSCC patients. Next-generation sequencing has become an increasingly valuable tool with widespread use in the oncology field and is being robustly explored for predicting rtAEs beyond dosimetric data.

**Methods:**

Patients who had Foundation Medicine sequencing data and received RT for primary or locally recurrent HNSCC were selected for this study. Early and late toxicity data were collected and reported based on Common Terminology Criteria for Adverse Events version 5.0. Dosimetric parameters were collected for pertinent structures.

**Results:**

A total of HNSCC 37 patients were analyzed in this study. Genetic alterations in BRCA2, ERBB3, NOTCH1 and CCND1 were all associated with higher mean grade of toxicity with BRCA2 alteration implicated in all toxicity parameters evaluated including mucositis, early dysphagia, xerostomia and to a lesser extent, late dysphagia. Interestingly, patients who exhibited alterations in both BRCA2 and ERBB3 experienced a twofold or greater increase in early dysphagia, early xerostomia and late dysphagia compared to ERBB3 alteration alone. Furthermore, several gene alterations were associated with improved toxicity outcomes. Within an RT supersensitive patient subset, alterations were found in TNFAIP3, HNF1A, SPTA1 and CASP8. All of these alterations were not found in the RT insensitive patient subset. We found 17 gene alterations in the RT insensitive patient subset that were not found in the RT supersensitive patient subset.

**Conclusion:**

Despite consistent RT dosimetric parameters, patients with HNSCC experience heterogeneous patterns of rtAEs. Identifying factors associated with toxicity outcomes offers a new avenue for personalized precision RT therapy and prophylactic management. Here, next-generation sequencing in a population of HNSCC patients correlates several genetic alterations with severity of rtAEs. Further analysis is urgently needed to identify genetic patterns associated with rtAEs in order to reduce harmful outcomes in this challenging population.

**Supplementary Information:**

The online version contains supplementary material available at 10.1186/s12967-021-02876-5.

## Background

Head and neck squamous cell carcinoma (HNSCC) is the 6th most prevalent cancer, resulting in 13,000 deaths annually in the United States alone [1]. Approximately 75% of HNSCC patients receive radiation therapy (RT) as standard of care for disease ranging from early to locally advanced [2]. Significant technological advancements including intensity modulated RT (IMRT) have allowed for meaningful reductions in dose to uninvolved organs at risk (OAR) [3–5]. Despite these innovations, RT toxicity continues to have a significant impact on patient recovery and quality of life, often resulting in delays or premature termination of treatment, which have both been associated with higher rates of local recurrence [6–8]. Specifically, missing two or more treatments has been associated with increased recurrence risk and inferior overall survival, with decrement to overall survival estimated at 1% per one missed day [9]. This is particularly consequential for human papillomavirus (HPV) negative patients with literature demonstrating higher likelihood of missed treatments compared to their HPV positive counterparts, which compounds with baseline inferior disease-related outcomes [10].

Dose limitations are uniquely challenging in HNSCC due to the proximity of critical OARs with early and late toxicity resulting in mucositis, dysphagia, xerostomia, tooth decay, vocal dysfunction and loss of taste [11]. While no studies have demonstrated a direct link between treatment-related toxicity and suicide in HNSCC, several reports including a surveillance, epidemiology, and end results (SEER) analysis of more than 300,000 head and neck cancer (HNC) patients have shown excessive rates of suicide in survivors of HNSCC; second only to survivors of pancreatic cancer [12, 13].

Current RT clinical recommendations are population-based with an underlying assumption that the patient population exhibits uniform RT sensitivity in normal tissue structures [14–21]. It is well established that there are interpatient discrepancies in toxicity outcomes that cannot be explained by dose alone. With the increasing use and affordability of gene sequencing, there is now abundant literature to suggest that gene alterations may play a significant role in a patient’s radiation response and subsequent RT outcome [19, 22]. This emerging evidence implicates gene modifications in critical biological pathways including DNA repair [21], cell cycle [23], stem cell regeneration [21], apoptosis [24] and immune responses [25–29], as well as molecular pathways involved in scavenging of reactive oxygen species (ROS) [30]. In a study of more than 400 breast cancer patients, Ambrosone et al. demonstrated that reduced activity of a glutathione S-transferase genotype resulted in a two-fold increase in acute skin toxicity when accounting for radiation dose [19]. Similar associations of specific germline variants and toxicity outcomes have been described in prostate and non-small cell lung cancer (NSCLC) [31].

Acknowledging the critical consequences associated with toxicity in HNSCC, there is compelling rationale to enhance our understanding of specific genetic factors associated with radiation therapy adverse events (rtAEs) in this population in order to develop personalized treatment regimens that serve to augment treatment adherence and quality of life. Here, we report an exploratory pilot study evaluating biomarkers in patients with HNSCC and report candidate genes as predictive of rtAEs.

## Methods

### Data source and patient selection

Thirty-seven HNSCC patients who underwent FoundationOne® CDx testing and received RT with available dosimetric data were selected for this study. FoundationOne® CDx is a FDA-approved tissue-based broad companion diagnostic (CDx) that is clinically and analytically validated for all solid tumors [32]. All selected patients in our study cohort (n = 37) were treated at the Moores Cancer Center at the University of California San Diego between 2014 and 2019.

### Patient Demographics and treatment variables

All patients received external beam radiation to a minimum dose of 28.5 Gray (Gy) (range 28.5–72, median 66 Gy, standard deviation (SD) 9.5 Gy). A total of three patients received less than 50 Gy. Potentially relevant patient and treatment characteristics were collected including age at diagnosis, gender, smoking history, HPV status and systemic therapy. Staging information was collected according to American Joint Committee on Cancer (AJCC) classification, 8th edition [33].

### Collection of toxicity data

Patient charts were utilized to report early and late rtAE endpoints for mucositis, dysphagia and xerostomia. Toxicities were recorded using Common Terminology Criteria for Adverse Events (CTCAE) grades of 1–5, which scored and reported by the treating physician on the day of service during therapy and in follow up. Early toxicity endpoints were recorded as the highest CTCAE grade experienced during therapy or within 6 weeks of completing therapy. Late toxicity endpoints were recorded as the highest CTCAE grade experienced from six months post-RT to the time of most recent follow up.

### Collection of dosimetric data

All patients underwent a computed-tomography (CT) simulation for RT treatment planning. Patients were fitted with an aquaplast mask for immobilization and CT was obtained with a 2.5 mm slice thickness. The following structures were delineated on all CT simulation scans: oral cavity, cricopharyngeus, parotid and submandibular glands (SMG) and posterior pharyngeal constrictors (PCM). The PCM was further divided into superior PCM, middle PCM and inferior PCM based on established contouring guidelines [34–36]. These structures were selected based on published literature supporting associations of these structures with the aforementioned rtAEs [37–39]. The mean dose to each of the delineated structures was then extracted based on each patient’s completed treatment course.

### Determination of patients who are supersensitive and insensitive to RT

Dose metrics were divided into quartiles and compared to toxicity outcomes. For a particular metric, patients were identified as ‘supersensitive’ if they received low dose, defined as a mean dose less than the 1st quartile dose value for the entire cohort, yet had poor outcomes (grade 2–5). Patients were identified as ‘insensitive’ if they received high dose, defined as a mean dose greater than the 3rd quartile dose value for the entire cohort, yet had good outcomes (grade 0–1). Patients who were supersensitive or insensitive for more than two structures were selected as overall supersensitive or insensitive for further genetic analysis.

### Statistical analysis

The gene alteration histogram was generated in R. Pearson Chi square tests were performed using SPSS V26.0 (SPSS. Inc.; Chicago, IL) to assess associations between genetic alterations and Grade 3 or higher toxicity.

## Results

### Patient characteristics

In our selected cohort of 37 patients, the median age was 65 (range 46–92) (Table 1). The majority of patients (67.5%) had stage III-IV HNSCC. Chemotherapy was given concurrently with RT in 86.4% of patients with regimens including cisplatin, cetuximab and pembrolizumab. Oral cavity was the most common primary site (43.2%) followed by oropharynx (21.6%) and larynx (16.2%) with 67.6% overall presenting with HPV negative disease. All patients received RT as part of their treatment course. The median radiation dose to the pharyngeal constrictor muscles and contralateral submandibular gland were 5172 cGy and 6643 cGy, respectively.

**Table 1:** Patient characteristics

Characteristic	Value	N = 37 (%)
Age	< 65	19 (51.4)
	≥ 65	18 (48.6)
Gender	Male	21 (56.8)
	Female	16 (43.2)
Smoking	< 10 pack years	21 (56.8)
	> 10 pack years	16 (43.2)
T Stage	1	7 (18.9)
	2	11 (29.7)
	3	6 (16.2)
	4	11 (29.7)
	Recurrent	2 (5.4)
N Stage	0	7 (18.9)
	1	8 (21.6)
	2	16 (43.2)
	3	4 (10.8)
	Recurrent	2 (5.4)
M Stage	0	36 (97.3)
	1	1 (2.7)
Overall Stage	I	6 (16.2)
	II	4 (10.8)
	II	7 (18.9)
	IV	18 (48.6)
	Recurrent	2 (5.4)
P 16 Status	Positive	12 (32.4)
	Negative	25 (67.6)
Primary Site	Oral Cavity	16 (43.2)
	Oropharynx	8 (21.6)
	Larynx	6 (16.2)
	Hypopharynx	4 (10.8)
	Nasopharynx	1 (2.7)
	Cutaneous	2 (5.4)

Toxicities are graded by CTCAE criteria

### Sequencing and toxicity analysis

**Fig. 1:**
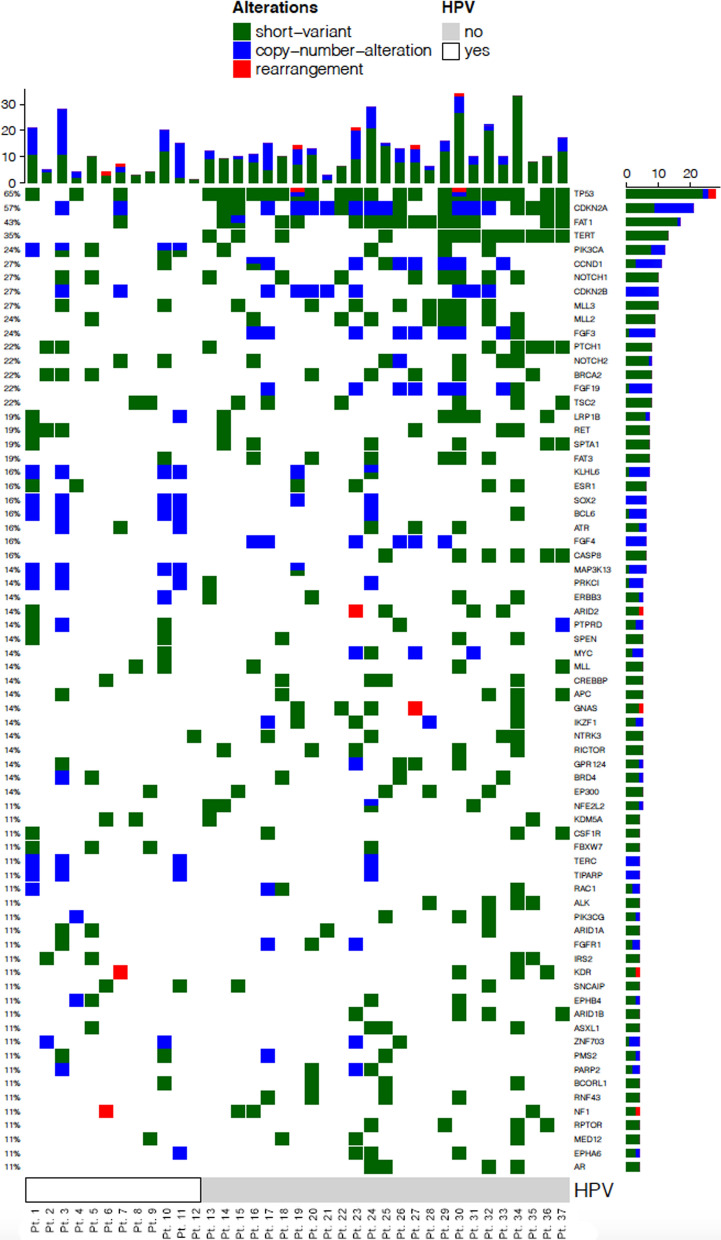
Top 71 commonly altered genes across 37 deidentified HNSCC samples. Samples are sorted by their HPV status. Alterations are color coded based on variant classification. Histogram on the x-axis indicates the number of gene alterations per sample. Histogram on the y-axis indicates the number of samples with the gene alteration

A histogram of the top 71 altered genes and associated alterations are shown (Fig. 1). The mean number of gene alterations in the cohort was 23. The most common alteration was TP53 (n = 24). Our study cohort compared well with published literature for HPV prevalence, as well as, known associations between gene variants and HPV status, including TP53, in head and neck cancer patients [40–46].

All patients experienced at least 1 rtAE reported as Grade 1 or higher based on CTCAE criteria. Approximately 62% of patients experienced a severe rtAE graded as 3 or higher based on CTCAE criteria. Early mucositis occurred in 89.2% of patients with approximately 35% experiencing severe mucositis. Late xerostomia and late dysphagia were similarly prevalent at 89.2% and 91.9%, respectively. While severe late dysphagia had similar rates to early mucositis (35%), severe late xerostomia was found in only 8.1% of patients. Early dysphagia occurred in 73% of patients with 18.9% experiencing severe symptoms. Importantly, in the five patients who received cetuximab, there was no association with severe mucositis (r = 0.165, p = 0.37) (Table 2).

**Table 2:** Toxicity outcomes

CTCAE grade	Early mucositis	Early dysphagia	Late xerostomia	Late dysphagia
	n (%)	n (%)	n (%)	n (%)
0	4 (10.8)	10 (27.0)	4 (10.8)	3 (8.1)
1	3 (8.1)	14 (40.5)	28 (75.7)	12 (32.4)
2	17 (45.9)	3 (8.1)	2 (5.4)	8 (21.6)
3	12 (32.4)	6 (16.2)	2 (5.4)	13 (35.1)
4	1 (2.7)	1 (2.7)	1 (2.7)	0 (0.0)

We assessed the correlations between the most prevalent gene alterations in our cohort and their related RT toxicities. In this evaluation, BRCA2, ERBB3, NOTCH1 and CCND1 genetic alterations correlated with increased mean grade of rtAEs at 27%, 38%, 5.72% and 8% respectively. Conversely, alterations in ATR, PIK3CA, CASP8, ESR1, and FAT1 appeared to show a protective overall effect on combined toxicity outcomes (Table 3). However, only BRCA2 alterations correlated with increased mean severity in all four clinical toxicity categories compared to patients with wild-type BRCA2: mucositis (31.82%) early dysphagia (45%), xerostomia (37%), and to a lesser extent late dysphagia (4.57%). Importantly, we found that some gene variants had a protective effect on rtAEs. PIK3CA alterations, for example, demonstrated improved toxicity outcomes in early dysphagia (p = 0.05). Notably, most gene variants showed variable outcomes on rtAEs. Mean toxicity, percent change and bivariate analysis of grade 3 or higher toxicity are shown in Table 3.

**Table 3:** Mean toxicities recorded for HNSCC patients (n = 37) grouped by presence of an alteration in a certain gene

Group	Early mucositis (mean)	Early dysphagia (mean)	Late xerostomia (mean)	Late dysphagia (mean)	Combined toxicity (mean)
BRCA2 Var (+) N = 8	2.5	1.75	1.38	1.88	7.5
BRCA2 Var (−) N = 29	1.9	1.21	1	1.79	5.9
% Change	31.82	45	37.5	4.57	27.19
Bivariate	p = 0.04	p = 0.07	p = 0.18	p = 0.74	p = 0.10
ATR Var (+) N = 6	2.5	1.17	0.83	1.5	6
ATR Var (−) N = 31	1.94	1.35	1.13	1.87	6.29
% Change	29.17	− 13.89	− 26.17	− 19.83	− 4.62
Bivariate	p = 0.05	p = 0.64	p = 0.30	p = 0.38	p = 0.81
ERBB3 Var (+) N = 5	2.6	2.4	1.4	1.8	8.2
ERBB3 Var (−) N = 32	1.94	1.16	1.03	1.81	5.94
% Change	34.19	107.57	35.76	− 0.69	38.11
Bivariate	p = 0.17	p = 0.05	p = 0.07	p = 0.71	p = 0.92
TP53 Var (+) N = 24	2.13	1.38	1.04	1.71	6.25
TP53 Var (−) N = 13	1.85	1.23	1.15	2	6.23
% Change	15.1	11.72	− 9.72	− 14.58	0.31
Bivariate	p = 0.87	p = 0.9	p = 0.46	p = 0.58	p = 0.04
NOTCH1 Var (+) N = 10	2.5	1.4	1.2	1.4	6.5
NOTCH1 Var (−) N = 27	1.85	1.3	1.04	1.96	6.15
% Change	35	8	15.71	− 28.68	5.72
Bivariate	p = 0.03	p = 0.72	p = 0.08	p = 0.34	p = 0.56
PIK3CA Var (+) N = 9	2.67	0.67	1.22	1.22	5.78
PIK3CA Var (−) N = 28	1.82	1.54	1.04	2	6.39
% Change	46.41	− 56.59	18.01	38.89	-9.62
Bivariate	p = 0.01	p = 0.05	p = 0.39	p = 0.02	p = 0.76
CDKN2A Var (+) N = 21	2	1.38	1	1.86	6.24
CDKN2A Var (−) N = 16	2.06	1.25	1.19	1.75	6.25
% Change	− 3.03	10.48	− 15.79	6.12	− 0.19
Bivariate	p = 0.90	p = 0.94	p = 0.88	p = 0.13	p = 0.97
CASP8 Var (+) N = 6	1.67	1	1	1.5	5.17
CASP8 Var (−) N = 31	2.1	1.36	1.1	1.87	6.45
% Change	− 20.51	− 26.67	− 8.82	− 19.83	− 19.92
Bivariate	p = 0.96	p = 0.64	p = 0.81	p = 0.96	p = 0.52
NFE2L2 Var (+) N = 4	2.5	1.6	1	1.5	6.25
NFE2L2 Var (−) N = 33	1.9	1.48	1.09	1.85	6.24
% Change	31.82	− 66.3	− 8.33	− 18.85	0.12
Bivariate	p = 0.04	p = 0.97	p = 0.49	p = 0.75	p = 0.10
CCND1 Var (+) N = 10	2.5	1	1	2.1	6.6
CCND1 Var (−) N = 27	1.9	1.57	1.07	1.7	6.11
% Change	31.82	− 36.36	2.41	23.26	8
Bivariate	p = 0.04	p = 0.64	p = 0.71	p = 0.56	p = 0.10
ESR1 Var (+) N = 6	2.5	1.33	0.83	0.83	4
ESR1 Var (−) N = 31	1.9	0.92	1.13	2	6.68
% Change	31.82	44.44	− 26.19	− 58.33	− 40.1
Bivariate	p = 0.04	p = 0.14	p = 0.30	p = 0.07	p = 0.01
FAT1 Var (+) N = 16	2.5	1.33	1	1.81	5.69
FAT1 Var (−) N = 21	1.9	0.92	1.14	1.81	6.67
% Change	31.82	44.44	− 12.5	0.16	− 14.69
Bivariate	p = 0.04	p = 0.06	p = 0.92	p = 0.71	p = 0.06

For each gene, the % change is calculated in relation to the altered (−) group. Toxicities are graded by CTCAE criteria

Given that BRCA2 variants were associated with uniform increase in rtAEs, the patients exhibiting BRCA2 alterations were paired with other prevalent gene alterations to assess their combined impact on rtAEs (Table 4). Interestingly, we find that BRCA2 and ERBB3 combination appeared to exhibit an additive effect on total rtAEs with a twofold increase or more in early dysphagia, early xerostomia and late dysphagia, compared to ERBB3 alteration alone.

**Table 4:** Mean toxicities recorded for HNSCC patients (n = 37) grouped by presence and absence of an alteration in certain genes

Group	Early mucositis (mean)	Early dysphagia (mean)	Late xerostomia (mean)	Late dysphagia (mean)	Combined toxicity (mean)
ATR (BRCA2 Var (+)) N = 4	2.5	1.25	0.75	1.5	6
ATR (BRCA2 Var (−)) N = 2	2.5	1	1	1.5	6
% Change	0	25	− 25	0	0
ERBB3 (BRCA2 Var (+)) N = 2	3	3.5	2	3	11.5
ERBB3 (BRCA2 Var (−)) N = 3	2.33	1.67	1	1	6
% Change	28.57	110	100	200	91.67
TP53 (BRCA2 Var (+)) N = 4	2.5	2	1.25	2	7.75
TP53 (BRCA2 Var (−)) N = 20	2.05	1.25	1	1.65	5.95
% Change	21.95	60	25	21.21	30.25
NOTCH1 (BRCA2 Var (+)) N = 4	2.5	1.5	1.5	2	7.5
NOTCH1 (BRCA2 Var (−)) N = 6	2.5	1.33	1	1	5.83
% Change	0	12.5	50	100	28.57
PIK3CA (BRCA2 Var (+)) N = 3	3	0.67	1.33	1	6
PIK3CA (BRCA2 Var (−)) N = 6	2.5	0.67	1.17	1.33	5.67
% Change	20	0	14.29	− 25	5.88
CDKN2A (BRCA2 Var (+)) N = 5	2.8	1.8	1.2	2	7.8
CDKN2A (BRCA2 Var (−)) N = 16	1.75	1.25	0.94	1.81	5.75
% Change	60	44	28	10.34	35.65
CCND1 (BRCA2 Var (+)) N = 2	2	2	1	2.5	7.5
CCND1 (BRCA2 Var (−)) N = 8	1.75	1.5	1.13	2	6.38
% Change	14.29	33.33	− 11.11	25	17.65
FAT1 (BRCA2 Var (+)) N = 3	2.33	1.33	0.67	1.67	6
FAT1 (BRCA2 Var (−)) N = 13	1.77	0.92	1.08	1.85	5.62
% Change	31.89	44.44	− 38.1	− 9.72	6.85

For each gene, the % change is calculated in respect to the BRCA2 (−) cohort. Toxicities are graded by CTCAE criteria

Similarly, although to a lesser extent, alterations including TP53, NOTCH1 and CDKN2A exhibited higher toxicity in the presence of BRCA2 variants.

### RT supersensitive and insensitive patients

To isolate contributing genetic factors from normal radiation dose response, the patient cohort was divided into three groups (normal, supersensitive, and insensitive response to radiation induced toxicities) based on the relationship between their normal tissue outcomes and the dose they received. Specifically, for a particular metric, patients were identified as supersensitive if they received low dose (defined as a mean dose less than the 1st quartile dose value for the entire cohort) yet had poor outcomes (grade 2–5). Patients were identified as insensitive if they received high dose (defined as a mean dose greater than the 3rd quartile dose value for the entire cohort) yet had good outcomes (grade 0–1). Patients who were supersensitive or insensitive for more than two structures were selected as overall supersensitive or insensitive for further genetic analysis. Note one patient was excluded from this analysis because their radiation was delivered in the form of a quadshot (higher dose per treatment, fewer treatments) and thus is expected to have a different biological effect than standard dose fractionation.

**Fig. 2:**
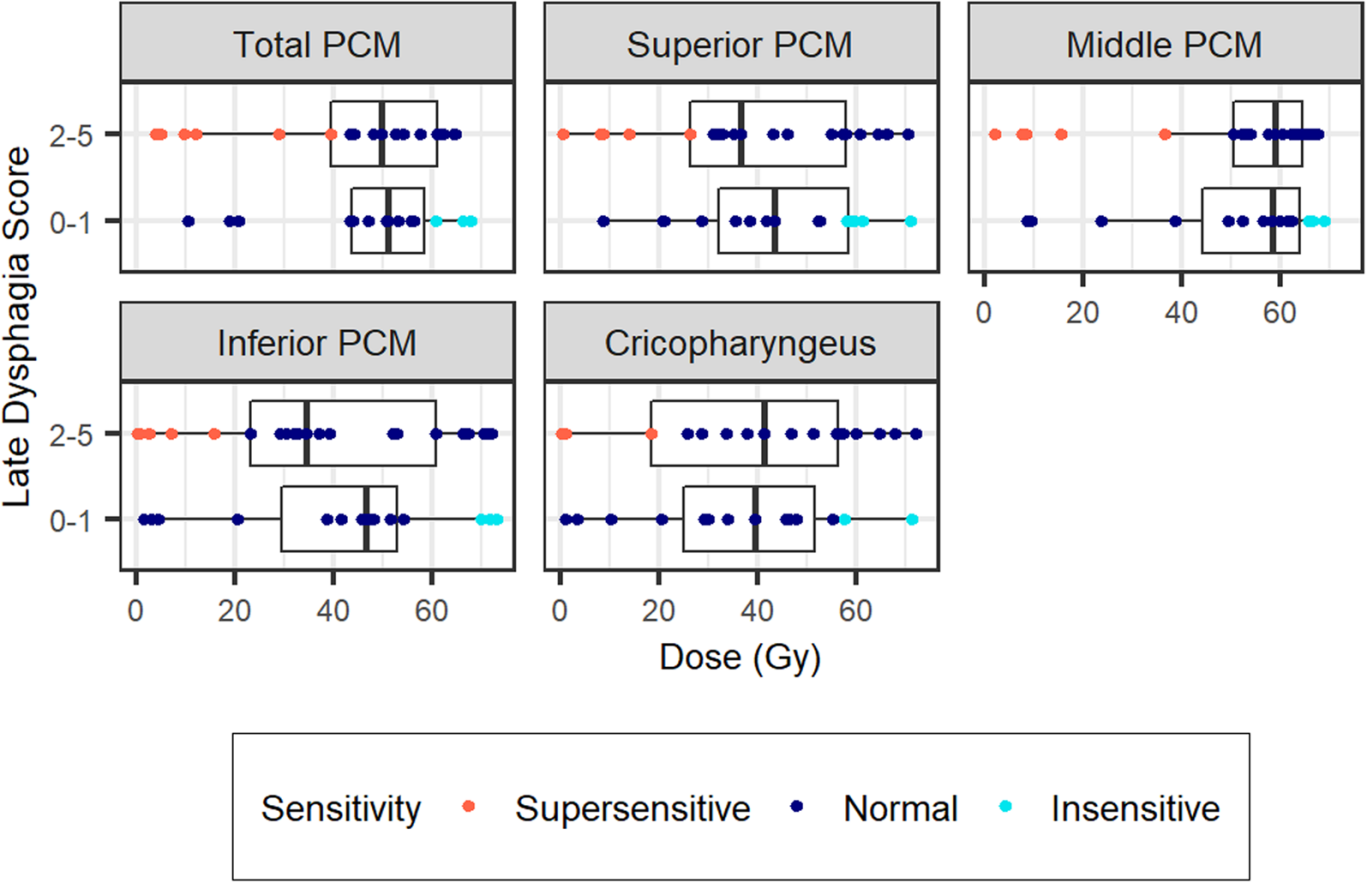
Mean doses to structures associated with late dysphagia versus late dysphagia outcomes for 36 patients are plotted. For a particular metric, patients were identified as supersensitive if they received low dose (defined as a mean dose less than the 1st quartile dose value for the entire cohort) yet had poor outcomes (grade 2–5). Patients were identified as insensitive if they received high dose (defined as a mean dose greater than the 3rd quartile dose value for the entire cohort) yet had good outcomes (grade 0–1). Patients who were supersensitive or insensitive for more than two structures were selected as overall supersensitive or insensitive for further genetic analysis

Based on the dose metrics, six patients were determined to be supersensitive and seven were determined to be insensitive to RT for late dysphagia (Fig. 2). All patients within both subsets had locally advanced disease. Upon evaluation of gene alterations, there were four gene alterations found in the supersensitive subset that were not present in the insensitive subset: TNFAIP3, HNF1A, SPTA1 and CASP8. All supersensitive patients were found to have an alteration in at least one of these aforementioned genes. None of the supersensitive patients were found to have BRCA2 gene alterations. Conversely, a total of 17 genetic alterations were found in the insensitive subset that were not found in any patients in the supersensitive subset (Table 5). Additionally, this same analysis was repeated for two other outcomes: early dysphagia and late xerostomia. However, for those outcomes, a strong dose dependence was observed and thus a reasonably sized subset of sensitive and insensitive patients could not be identified, figures seen in Additional file 1. A larger cohort may be needed to investigate these outcomes separately.

**Table 5:** Differentially altered genes found between patients supersensitive and insensitive to RT

Gene	% of Gene variant in group A (supersensitive, n = 6)	% of Gene variant in group C (insensitive, n = 7)
CDKN2A	83.33	42.86
FAT1	66.67	28.57
TNFAIP3	33.33	0
HNF1A	33.33	0
SPTA1	33.33	0
CASP8	33.33	0
NOTCH2	33.33	14.29
MAP3K1	33.33	14.29
KLHL6	0	42.86
MAP3K13	0	42.86
SOX2	0	42.86
BCL6	0	28.57
BRCA2	0	28.57
BRD4	0	28.57
ESR1	0	28.57
GPR124	0	28.57
GSK3B	0	28.57
MLL3	0	28.57
PIK3CB	0	28.57
PRKCI	0	28.57
SNCAIP	0	28.57
SUFU	0	28.57
TERC	0	28.57
TIPARP	0	28.57
ZNF703	0	28.57

## Discussion

The utilization of next-generation sequencing in cancer patients offers tremendous potential to predict gene alterations associated with treatment-related toxicities that cannot be fully accounted for by treatment parameters alone. Despite this readily available resource, the literature on predictive biomarkers of RT toxicity remains limited with the majority of genomic studies focusing on treatment response and targeted therapies rather than toxicity [47, 48]. Our findings join the ranks of studies correlating distinct genetic alterations with rtAEs in cancer patients [23, 26, 42]. Here, we report the first analysis to date that associates genetic alterations in HNSCC with rtAEs. These novel findings provide a vital springboard for the development of predictive tools that can be rapidly translated into clinical practice.

As a benchmark for assessment, patients were initially evaluated based on combined toxicity profiles. While we believe these findings are helpful in establishing correlation with gene alterations, it was also critically important to separate toxicities that likely have different underlying cellular mechanisms. Broadly speaking, early rtAEs are generally associated with cellular injury leading to cellular depletion and local inflammation [49]. Infiltration of innate immune cells and subsequent release of cytokines including tumor necrosis factor (TNF), interleukin (IL)-1 and IL-6 have all been implicated in this process [50]. Conversely, late rtAEs have largely been attributed to fibrosis and atrophy with associations with transforming growth factors (TGF), IL-6, and TNF alpha (TNFα) [51, 52]. It is, therefore, unsurprising that consequential gene alterations will vary significantly by the type of rtAE being assessed.

In our population, for example, BRCA2 alteration was observed to result in a 32% and 45% increase in the mean toxicity grade for early mucositis and early dysphagia, respectively, with only a 5% difference seen in late dysphagia (Table 3). Further separating BRCA2 variants from late dysphagia was our finding that patients who were deemed supersensitive to RT experienced severe late dysphagia at a rate of 50% with a mean toxicity grade of 2.5; notably with no BRCA2 alterations seen in this population. This is in contrast to a mean toxicity grade of 1.88 for late dysphagia observed in the population with BRCA alterations. This aligns with previous literature of BRCA2 variant carriers receiving RT for breast cancer in which no increase in late toxicity was observed, though the comparison is imperfect given anatomical differences and RT dose rarely exceed 50 Gy in breast RT [53, 54]. While these findings are preliminary, they serve to emphasize that there are unique underlying cellular mechanisms for toxicity parameters that likely associate with individual genomic signatures.

The distinct underlying mechanisms associated with toxicity parameters also creates the possibility that gene alterations may be both protective and harmful depending on the outcome being evaluated. In our cohort, the mean grade of early dysphagia was 44% higher in patients with ESR1 alteration, but mean grade of late dysphagia was 58% lower in this population. Similar discordant findings between early and late toxicities were observed for PIK3CA, NOTCH1 and CCND1 (Table 3).

In our analysis combining BRCA2 alterations with other frequently altered genes, we observed synergistic augmentation in rtAEs, particularly with ERBB3 (Table 4). This serves to highlight the value of generating genetic risk profiles that incorporate comprehensive genomic data in order to further stratify a patient’s individual risk. A previous prospective analysis of the association of gene alterations and rtAEs in breast and prostate cancer patients failed to validate previously published findings of individual associated genes, but emphasized the importance of generating more robust radiogenomic databases to elucidate the value of genetic risk profiles [17].

Remarkably, we find that somatic tumor gene alterations influence surrounding normal tissue. How this influences damage to healthy tissues in response to RT is currently unknown. We can hypothesize that this effect is mediated by release of cytokines, as well as, the immune environment of the cancer. For example, ERBB3 activation could be related to signal transduction pathways that influence inflammatory mediators, among them, cytokines and chemokines.

Especially compelling results in our analysis were the differential gene alterations found between patients deemed supersensitive and insensitive to RT, with several genes notably involved in inflammatory and apoptotic pathways (Table 5). TNFAIP3, for example, was altered in 33% of supersensitive patients but 0% of insensitive patients. This gene encodes proteins involved in cytokine-mediated immune and inflammatory responses including modulation of NF-kB and TNF-mediated apoptosis [55–58]. In the insensitive subset, nearly 43% of patients harbored an alteration in MAP3K13, which is involved in proliferation and apoptosis via the JNK signaling pathway. This alteration was not found in any patients in the supersensitive subset.

Providing physicians with a tool that identifies these supersensitive and insensitive patients could guide their decisions regarding treatment. In particular, supersensitive patients could be recommended for frequent adaptive radiotherapy in order to minimize the absolute dose received to their normal tissue structures. This strategy would ensure that adaptive radiotherapy, which requires extra clinical resources, is directed at the patients likely to receive the greatest benefit and in turn potentially improve outcomes for these patients. For insensitive patients, physicians could feel more confident in prioritizing tumor dose coverage over sparing of adjacent normal tissues. This is a trade-off that is considered for almost all head-and-neck radiotherapy treatment plans and thus the biomarkers demonstrated here could be immensely beneficial for clinical decision-making.

## Conclusions

In summary, our findings present an analysis of the relationship of gene alterations with rtAEs in HNSCC utilizing next-generation sequencing. We find that somatic tumor gene alterations influence damage to healthy tissues in response to RT. In addition, our data suggests that rtAEs cannot be consistently predicted by a single gene alteration, which is consistent with current thinking in the field [21]. Despite prior studies associating distinct genes with rtAEs, there is very limited literature incorporating multiplex panels to generate genetic risk profiles. Therefore, we believe these findings highlight the urgent need to expand genomic analyses in this patient population with a goal of establishing genetic risk profiles and ultimately guide therapeutic regimens that optimize the therapeutic ratio.

## Supplementary Information

**Additional file 1: Figure S1.** Early Dysphagia by RT Sensitivity.Mean doses to structures associated with earlydysphagia versus earlydysphagia outcomes for 36 patients are plotted. For a particular metric, patients were identified as sensitive if they received low dose (defined as a mean dose less than the 1st quartile dose value for the entire cohort) yet had poor outcomes (grade 2–5). Patients were identified as insensitive if they received high dose (defined as a mean dose greater than the 3rd quartile dose value for the entire cohort) yet had good outcomes (grade 0–1). Patients who were supersensitive or insensitive for more than two structures were selected as overall supersensitive or insensitive for further genetic analysis. Only 1 patient was identified as overall supersensitive and 8 patients were identified as overall insensitive. Thus a larger overall cohort is needed to investigate genetic differences between supersensitive and insensitive patients for this outcome. **Figure S2.** Late Xerostomia by RT Sensitivity. Mean doses to structures associated with late xerostomia versus late xerostomia outcomes for 36 patients are plotted. For a particular metric, patients were identified as sensitive if they received low dose (defined as a mean dose less than the 1st quartile dose value for the entire cohort) yet had poor outcomes (grade 2–5). Patients were identified as insensitive if they received high dose (defined as a mean dose greater than the 3rd quartile dose value for the entire cohort) yet had good outcomes (grade 0–1). Patients who were supersensitive or insensitive for more than two structures were selected as overall supersensitive or insensitive for further genetic analysis. Only 1 patient was identified as overall supersensitive while 2 patients were identified as overall insensitive. Thus a larger overall cohort is needed to investigate genetic differences between supersensitive and insensitive patients for this outcome. **Table S1.** Differentially altered genes. **Table S2.** Supersensitive group gene variants. **Table S3.** Gene variants.

## Data Availability

The datasets used, generated, and analyzed during the current study are available from the corresponding author on reasonable request.

## Abbreviations

AJCC: American Joint Committee on Cancer
ATR: ATR serine/threonine kinase
BCL6: BCL6 transcription repressor
BRCA2: Breast cancer 2 (BRCA2) DNA repair associated
BRD4: Bromodomain containing 4
CASP8: Caspase 8
CCND1: Cyclin D1
CDKN2A: Cyclin dependent kinase inhibitor 2A
CT: Computed-tomography
CTCAE: Common Terminology Criteria for Adverse Events
ERBB3: Erb-b2 receptor tyrosine kinase 3
ESR1: Estrogen receptor 1
FAT1: AT atypical cadherin 1
GPR124: Adhesion G protein-coupled receptor A2
Gy: Gray
HNC: Head and neck cancer
HNF1A: HNF1 homeobox A
HNSCC: Head and neck squamous cell carcinoma
HPV: Human papillomavirus
IGRT: Image-guided RT
IL: Interleukin
IMRT: Intensity modulated RT
KLHL6: Kelch like family member 6
MAP3K1: Mitogen-activated protein kinase kinase kinase 1
MAP3K13: Mitogen-activated protein kinase kinase kinase 13
MLL3: Lysine methyltransferase 2C
NOTCH1: Notch receptor 1
NOTCH2: Notch receptor 2
NSCLC: Non-small cell lung cancer
OAR: Organs at risk
PCM: Posterior pharyngeal constrictors
PIK3CA: Phosphatidylinositol-4,5-bisphosphate 3-kinase catalytic subunit alpha
ROS: Reactive oxygen species
RT: Radiation therapy
rtAEs: Radiation therapy adverse events
SD: Standard deviation
SEER: Surveillance, epidemiology, and end results
SMG: Submandibular glands
SNCAIP: Synuclein alpha interacting protein
SOX2: Sex determing region Y (SRY)-box transcription factor 2
SPTA1: Spectrin alpha, erythrocytic 1
TERC: Telomerase RNA component
TGF: Transforming growth factors
TIPARP: TCDD inducible poly(ADP-ribose) polymerase
TNF: Tumor necrosis factor
TNFAIP3: Tumor necrosis factor, alpha-induced protein 3
TP53: Tumor protein 53
ZNF703: Zinc finger protein 703

## References

[1] Siegel RL, Miller KD, Jemal A (2019). Cancer statistics, 2019. CA Cancer J Clin.

[2] Ratko TA, Douglas GW, de Souza JA, Belinson SE, Aronson N. Radiotherapy treatments for head and neck cancer update. 2014. 25590120

[3] Baumann M, Krause M, Overgaard J, Debus J, Bentzen SM, Daartz J (2016). Radiation oncology in the era of precision medicine. Nat Rev Cancer.

[4] Lee J-H, Lee JCW, Leung W, Li M, Constant K, Chan CT (2008). Polarization Engineering Of Thermal Radiation Using Metallic Photonic Crystals. Adv Mater.

[5] Nutting CM, Morden JP, Harrington KJ, Urbano TG, Bhide SA, Clark C (2011). Parotid-sparing intensity modulated versus conventional radiotherapy in head and neck cancer (PARSPORT): a phase 3 multicentre randomised controlled trial. Lancet Oncol.

[6] Ghanem AI, Schymick M, Bachiri S, Mannari A, Sheqwara J, Burmeister C (2019). The effect of treatment package time in head and neck cancer patients treated with adjuvant radiotherapy and concurrent systemic therapy. World J Otorhinolaryngol Head Neck Surg.

[7] Suwinski R, Sowa A, Rutkowski T, Wydmanski J, Tarnawski R, Maciejewski B (2003). Time factor in postoperative radiotherapy: a multivariate locoregional control analysis in 868 patients. Int J Radiat Oncol Biol Phys.

[8] Langendijk JA, de Jong MA, Leemans CR, de Bree R, Smeele LE, Doornaert P (2003). Postoperative radiotherapy in squamous cell carcinoma of the oral cavity: the importance of the overall treatment time. Int J Radiat Oncol Biol Phys.

[9] Ohri N, Rapkin BD, Guha C, Kalnicki S, Garg M (2016). Radiation therapy noncompliance and clinical outcomes in an urban academic cancer center. Int J Radiat Oncol Biol Phys.

[10] Hess CB, Rash DL, Daly ME, Farwell DG, Bishop J, Vaughan AT (2014). Competing causes of death and medical comorbidities among patients with human papillomavirus-positive vs human papillomavirus-negative oropharyngeal carcinoma and impact on adherence to radiotherapy. JAMA Otolaryngol Head Neck Surg.

[11] Siddiqui F, Movsas B (2017). Management of radiation toxicity in head and neck cancers. Semin Radiat Oncol.

[12] Osazuwa-Peters N, Simpson MC, Zhao L, Boakye EA, Olomukoro SI, Deshields T (2018). Suicide risk among cancer survivors: head and neck versus other cancers. Cancer.

[13] Zeller JL. High suicide risk found for patients with head and neck cancer. JAMA. 296. United States; 2006. p. 1716–7. 10.1001/jama.296.14.1716 17032977

[14] West CM, Barnett GC (2011). Genetics and genomics of radiotherapy toxicity: towards prediction. Genome Med.

[15] Kelsey CR, Rosenstein BS, Marks LB (2012). Predicting toxicity from radiation therapy–it's genetic, right?. Cancer.

[16] Popanda O, Marquardt JU, Chang-Claude J, Schmezer P (2009). Genetic variation in normal tissue toxicity induced by ionizing radiation. Mutat Res.

[17] Barnett GC, Coles CE, Elliott RM, Baynes C, Luccarini C, Conroy D (2012). Independent validation of genes and polymorphisms reported to be associated with radiation toxicity: a prospective analysis study. Lancet Oncol.

[18] Svensson JP, Stalpers LJ, Esveldt-van Lange RE, Franken NA, Haveman J, Klein B (2006). Analysis of gene expression using gene sets discriminates cancer patients with and without late radiation toxicity. PLoS Med..

[19] Ambrosone CB, Tian C, Ahn J, Kropp S, Helmbold I, von Fournier D (2006). Genetic predictors of acute toxicities related to radiation therapy following lumpectomy for breast cancer: a case-series study. Breast Cancer Res.

[20] Rieger KE, Hong WJ, Tusher VG, Tang J, Tibshirani R, Chu G (2004). Toxicity from radiation therapy associated with abnormal transcriptional responses to DNA damage. Proc Natl Acad Sci U S A.

[21] Bergom C, West CM, Higginson DS, Abazeed ME, Arun B, Bentzen SM (2019). The implications of genetic testing on radiation therapy decisions: a guide for radiation oncologists. Int J Radiat Oncol Biol Phys.

[22] Kerns SL, Ostrer H, Stock R, Li W, Moore J, Pearlman A (2010). Genome-wide association study to identify single nucleotide polymorphisms (SNPs) associated with the development of erectile dysfunction in African-American men after radiotherapy for prostate cancer. Int J Radiat Oncol Biol Phys.

[23] Finnon P, Robertson N, Dziwura S, Raffy C, Zhang W, Ainsbury L (2008). Evidence for significant heritability of apoptotic and cell cycle responses to ionising radiation. Hum Genet.

[24] Schmitz A, Bayer J, Dechamps N, Goldin L, Thomas G (2007). Heritability of susceptibility to ionizing radiation-induced apoptosis of human lymphocyte subpopulations. Int J Radiat Oncol Biol Phys.

[25] Taylor AM, Groom A, Byrd PJ (2004). Ataxia-telangiectasia-like disorder (ATLD)-its clinical presentation and molecular basis. DNA Repair (Amst).

[26] O'Driscoll M, Cerosaletti KM, Girard PM, Dai Y, Stumm M, Kysela B (2001). DNA ligase IV mutations identified in patients exhibiting developmental delay and immunodeficiency. Mol Cell.

[27] Jyonouchi S, Orange J, Sullivan KE, Krantz I, Deardorff M (2013). Immunologic features of Cornelia de Lange syndrome. Pediatrics.

[28] Dutrannoy V, Demuth I, Baumann U, Schindler D, Konrat K, Neitzel H (2010). Clinical variability and novel mutations in the NHEJ1 gene in patients with a Nijmegen breakage syndrome-like phenotype. Hum Mutat.

[29] Chavoshzadeh Z, Hashemitari A, Darougar S (2018). Neurological manifestations of primary immunodeficiencies. Iran J Child Neurol.

[30] Zhao W, Diz DI, Robbins ME (2007). Oxidative damage pathways in relation to normal tissue injury. Br J Radiol..

[31] Rosenstein BS (2017). Radiogenomics: identification of genomic predictors for radiation toxicity. Semin Radiat Oncol.

[32] Foundation Medicine I. FoundationOne ® CDx. https://www.foundationmedicine.com/test/foundationone-cdx.

[33] Amin MB, Greene FL, Edge SB, Compton CC, Gershenwald JE, Brookland RK (2017). The Eighth Edition AJCC Cancer Staging Manual: Continuing to build a bridge from a population-based to a more "personalized" approach to cancer staging. CA Cancer J Clin..

[34] Schwartz DL, Hutcheson K, Barringer D, Tucker SL, Kies M, Holsinger FC (2010). Candidate dosimetric predictors of long-term swallowing dysfunction after oropharyngeal intensity-modulated radiotherapy. Int J Radiat Oncol Biol Phys.

[35] Christianen ME, Langendijk JA, Westerlaan HE, van de Water TA, Bijl HP (2011). Delineation of organs at risk involved in swallowing for radiotherapy treatment planning. Radiother Oncol.

[36] Brouwer CL, Steenbakkers RJ, Bourhis J, Budach W, Grau C, Grégoire V (2015). CT-based delineation of organs at risk in the head and neck region: DAHANCA, EORTC, GORTEC, HKNPCSG, NCIC CTG, NCRI, NRG Oncology and TROG consensus guidelines. Radiother Oncol.

[37] Mazzola R, Ricchetti F, Fiorentino A, Fersino S, Giaj Levra N, Naccarato S (2014). Dose-volume-related dysphagia after constrictor muscles definition in head and neck cancer intensity-modulated radiation treatment. Br J Radiol.

[38] Caudell JJ, Schaner PE, Desmond RA, Meredith RF, Spencer SA, Bonner JA (2010). Dosimetric factors associated with long-term dysphagia after definitive radiotherapy for squamous cell carcinoma of the head and neck. Int J Radiat Oncol Biol Phys.

[39] Cheng SC, Wu VW, Kwong DL, Ying MT (2011). Assessment of post-radiotherapy salivary glands. Br J Radiol.

[40] Vogelstein B, Lane D, Levine AJ (2000). Surfing the p53 network. Nature.

[41] Guimaraes DP, Hainaut P (2002). TP53: a key gene in human cancer. Biochimie.

[42] Gasco M, Crook T (2003). The p53 network in head and neck cancer. Oral Oncol.

[43] Poeta ML, Manola J, Goldwasser MA, Forastiere A, Benoit N, Califano JA (2007). TP53 mutations and survival in squamous-cell carcinoma of the head and neck. N Engl J Med.

[44] Mundi N, Prokopec SD, Ghasemi F, Warner A, Patel K, MacNeil D (2019). Genomic and human papillomavirus profiling of an oral cancer cohort identifies TP53 as a predictor of overall survival. Cancers Head Neck.

[45] Gross AM, Orosco RK, Shen JP, Egloff AM, Carter H, Hofree M (2014). Multi-tiered genomic analysis of head and neck cancer ties TP53 mutation to 3p loss. Nat Genet.

[46] Neskey DM, Osman AA, Ow TJ, Katsonis P, McDonald T, Hicks SC (2015). Evolutionary action score of TP53 identifies high-risk mutations associated with decreased survival and increased distant metastases in head and neck cancer. Cancer Res.

[47] Cho J, Johnson DE, Grandis JR (2018). Therapeutic implications of the genetic landscape of head and neck cancer. Semin Radiat Oncol.

[48] Riaz N, Morris LG, Lee W, Chan TA (2014). Unraveling the molecular genetics of head and neck cancer through genome-wide approaches. Genes Dis.

[49] King SN, Dunlap NE, Tennant PA, Pitts T (2016). Pathophysiology of radiation-induced dysphagia in head and neck cancer. Dysphagia.

[50] Dörr W (2003). Modulation of repopulation processes in oral mucosa: experimental results. Int J Radiat Biol.

[51] Remy J, Wegrowski J, Crechet F, Martin M, Daburon F (1991). Long-term overproduction of collagen in radiation-induced fibrosis. Radiat Res.

[52] Straub JM, New J, Hamilton CD, Lominska C, Shnayder Y, Thomas SM (2015). Radiation-induced fibrosis: mechanisms and implications for therapy. J Cancer Res Clin Oncol.

[53] Shanley S, McReynolds K, Ardern-Jones A, Ahern R, Fernando I, Yarnold J (2006). Late toxicity is not increased in BRCA1/BRCA2 mutation carriers undergoing breast radiotherapy in the United Kingdom. Clin Cancer Res.

[54] Pierce LJ, Haffty BG (2011). Radiotherapy in the treatment of hereditary breast cancer. Semin Radiat Oncol.

[55] Dixit VM, Green S, Sarma V, Holzman LB, Wolf FW, O'Rourke K (1990). Tumor necrosis factor-alpha induction of novel gene products in human endothelial cells including a macrophage-specific chemotaxin. J Biol Chem.

[56] Opipari AW, Boguski MS, Dixit VM (1990). The A20 cDNA induced by tumor necrosis factor alpha encodes a novel type of zinc finger protein. J Biol Chem.

[57] Beyaert R, Heyninck K, Van Huffel S (2000). A20 and A20-binding proteins as cellular inhibitors of nuclear factor-kappa B-dependent gene expression and apoptosis. Biochem Pharmacol.

[58] Catrysse L, Vereecke L, Beyaert R, van Loo G (2014). A20 in inflammation and autoimmunity. Trends Immunol.

