# Supplementary material for: Gene alterations as predictors of radiation-induced toxicity in head and neck squamous cell carcinoma
Source: J Transl Med. 2021 May 17;19:212. doi: 10.1186/s12967-021-02876-5 (PMC8130372; doi:10.1186/s12967-021-02876-5)
Supplement: Supplementary file 1 — Additional file 1: Figure S1. Early Dysphagia by RT Sensitivity.Mean doses to structures associated with earlydysphagia versus earlydysphagia outcomes for 36 patients are plotted. For a particular metric, patients were identified as sensitive if they received low dose (defined as a mean dose less than the 1st quartile dose value for the entire cohort) yet had poor outcomes (grade 2–5). Patients were identified as insensitive if they received high dose (defined as a mean dose greater than the 3rd quartile dose value for the entire cohort) yet had good outcomes (grade 0–1). Patients who were supersensitive or insensitive for more than two structures were selected as overall supersensitive or insensitive for further genetic analysis. Only 1 patient was identified as overall supersensitive and 8 patients were identified as overall insensitive. Thus a larger overall cohort is needed to investigate genetic differences between supersensitive and insensitive patients for this outcome. Figure S2. Late Xerostomia by RT Sensitivity. Mean doses to structures associated with late xerostomia versus late xerostomia outcomes for 36 patients are plotted. For a particular metric, patients were identified as sensitive if they received low dose (defined as a mean dose less than the 1st quartile dose value for the entire cohort) yet had poor outcomes (grade 2–5). Patients were identified as insensitive if they received high dose (defined as a mean dose greater than the 3rd quartile dose value for the entire cohort) yet had good outcomes (grade 0–1). Patients who were supersensitive or insensitive for more than two structures were selected as overall supersensitive or insensitive for further genetic analysis. Only 1 patient was identified as overall supersensitive while 2 patients were identified as overall insensitive. Thus a larger overall cohort is needed to investigate genetic differences between supersensitive and insensitive patients for this outcome. Table S1. Differentially alter [file 12967_2021_2876_MOESM1_ESM.pdf]

Supplementary Figures.

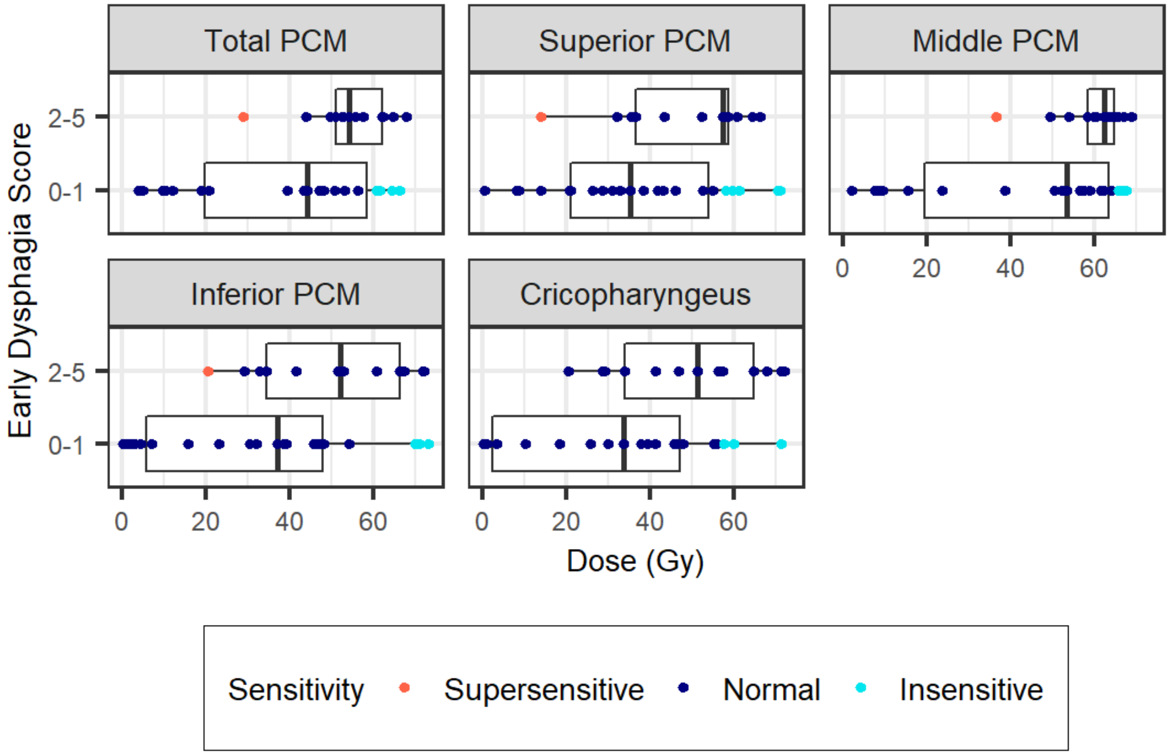

Figure S1. Early Dysphagia by RT Sensitivity. Mean doses to structures associated with early dysphagia versus early dysphagia outcomes for 36 patients are plotted. For a particular metric, patients were identified as sensitive if they received low dose (defined as a mean dose less than the 1st quartile dose value for the entire cohort) yet had poor outcomes (grade 2-5). Patients were identified as insensitive if they received high dose (defined as a mean dose greater than the 3rd quartile dose value for the entire cohort) yet had good outcomes (grade 0-1). Patients who were supersensitive or insensitive for more than two structures were selected as overall supersensitive or insensitive for further genetic analysis.Only 1 patient was identified as overall supersensitive and 8 patients were identified as overall insensitive. Thus a larger overall cohort is needed to investigate genetic differences between supersensitive and insensitive patients for this outcome.

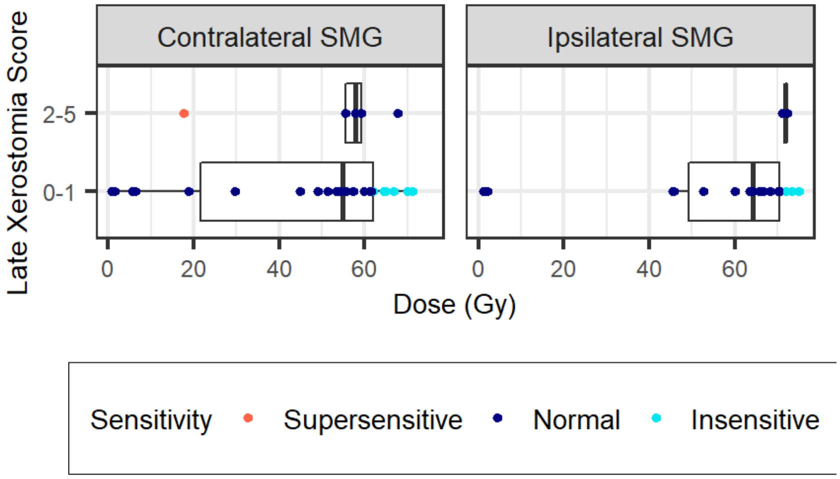

Figure S2. Late Xerostomia by RT Sensitivity. Mean doses to structures associated with late xerostomia versus late xerostomia outcomes for 36 patients are plotted. For a particular metric, patients were identified as sensitive if they received low dose (defined as a mean dose less than the 1st quartile dose value for the entire cohort) yet had poor outcomes (grade 2-5). Patients were identified as insensitive if they received high dose (defined as a mean dose greater than the 3rd quartile dose value for the entire cohort) yet had good outcomes (grade 0-1). Patients who were supersensitive or insensitive for more than two structures were selected as overall supersensitive or insensitive for further genetic analysis. Only 1 patient was identified as overall supersensitive while 2 patients were identified as overall insensitive. Thus a larger overall cohort is needed to investigate genetic differences between supersensitive and insensitive patients for this outcome.

Supplementary Tables.

Table S1. Differentially altered genes.

Gene	% Mutation in Group A (supersensitive, n=6)	% Mutation in Group C (Insensitive, n=7)	% Mutation in remaining Cohort (n=24)
BRCA2	0	28.6	25.81
ATR	16.7	28.6	12.5
ERBB3	0	0	20.8
TP53	50	42.9	75
NOTCH1	0	14.3	37.5
PIK3CA	0	28.6	25
CDKN2A	83.3	42.9	54.2
CASP8	33.3	0	16.7
NFE2L2	16.7	0	12.5
CCND1	16.7	14.3	33.3
ESR1	0	28.6	16.6
FAT1	66.7	28.6	37.5
**Group**	**% HPV Positive**		
A (supersensitive, n=6)	33.33		
C (insensitive, n=7)	71.43		

Table S2. Supersensitive group gene variants.

Gene	Group A (n=6) Supersensitive	Remaining Cohort (n=31)	Diff
TNFAIP3	33.33333333	0	33.33333333
MLL3	0	32.25806452	32.25806452
NOTCH1	0	32.25806452	32.25806452
CDKN2A	83.33333333	51.61290323	31.72043011
HNF1A	33.33333333	3.225806452	30.10752688
MAP3K1	33.33333333	3.225806452	30.10752688
FGF3	0	29.03225806	29.03225806
MLL2	0	29.03225806	29.03225806
FAT1	66.66666667	38.70967742	27.95698925
BRCA2	0	25.80645161	25.80645161
FGF19	0	25.80645161	25.80645161
FAT3	0	22.58064516	22.58064516
CASP8	33.33333333	12.90322581	20.43010753
BCL6	0	19.35483871	19.35483871
ESR1	0	19.35483871	19.35483871
FGF4	0	19.35483871	19.35483871
KLHL6	0	19.35483871	19.35483871
SOX2	0	19.35483871	19.35483871
TP53	50	67.74193548	17.74193548
SPTA1	33.33333333	16.12903226	17.20430108
DDR2	16.66666667	0	16.66666667
FANCA	16.66666667	0	16.66666667
FANCI	16.66666667	0	16.66666667
FGF23	16.66666667	0	16.66666667
GEN1	16.66666667	0	16.66666667
HRAS	16.66666667	0	16.66666667
HSP90AA1	16.66666667	0	16.66666667
PARP1	16.66666667	0	16.66666667
REL	16.66666667	0	16.66666667
SOCS1	16.66666667	0	16.66666667
TNKS2	16.66666667	0	16.66666667
BRD4	0	16.12903226	16.12903226
ERBB3	0	16.12903226	16.12903226
GNAS	0	16.12903226	16.12903226
GPR124	0	16.12903226	16.12903226
IKZF1	0	16.12903226	16.12903226
MAP3K13	0	16.12903226	16.12903226
MYC	0	16.12903226	16.12903226
PRKCI	0	16.12903226	16.12903226
SPEN	0	16.12903226	16.12903226
NOTCH2	33.33333333	19.35483871	13.97849462
AKT2	16.66666667	3.225806452	13.44086022
BCOR	16.66666667	3.225806452	13.44086022
BLM	16.66666667	3.225806452	13.44086022
FAM46C	16.66666667	3.225806452	13.44086022
MEN1	16.66666667	3.225806452	13.44086022
MKNK1	16.66666667	3.225806452	13.44086022
MST1R	16.66666667	3.225806452	13.44086022
ALK	0	12.90322581	12.90322581
EPHA6	0	12.90322581	12.90322581
EPHB4	0	12.90322581	12.90322581
FBXW7	0	12.90322581	12.90322581
FGFR1	0	12.90322581	12.90322581
IRS2	0	12.90322581	12.90322581
KDM5A	0	12.90322581	12.90322581
MED12	0	12.90322581	12.90322581
NF1	0	12.90322581	12.90322581
PARP2	0	12.90322581	12.90322581
RAC1	0	12.90322581	12.90322581
RPTOR	0	12.90322581	12.90322581
SNCAIP	0	12.90322581	12.90322581
TERC	0	12.90322581	12.90322581
TIPARP	0	12.90322581	12.90322581
ZNF703	0	12.90322581	12.90322581
CCND1	16.66666667	29.03225806	12.3655914

Table S3. Gene variants.

Gene	Group A (n=6)	Group C (n=7)
	Supersensitive	Insensitive
KLHL6	0	42.85714286
MAP3K13	0	42.85714286
SOX2	0	42.85714286
CDKN2A	83.33333333	42.85714286
HPV	33.33333333	71.42857143
FAT1	66.66666666	28.57142857
CASP8	33.33333333	0
HNF1A	33.33333333	0
SPTA1	33.33333333	0
TERT	33.33333333	0
TNFAIP3	33.33333333	0
BCL6	0	28.57142857
BRCA2	0	28.57142857
BRD4	0	28.57142857
ESR1	0	28.57142857
GPR124	0	28.57142857
GSK3B	0	28.57142857
MLL3	0	28.57142857
PIK3CB	0	28.57142857
PRKCI	0	28.57142857
SNCAIP	0	28.57142857
SUFU	0	28.57142857
TERC	0	28.57142857
TIPARP	0	28.57142857
ZNF703	0	28.57142857
MAP3K13	33.33333333	14.28571429
NOTCH2	33.33333333	14.28571429
AKT2	16.66666667	0
AR	16.66666667	0
ARID1B	16.66666667	0
ARID2	16.66666667	0
ASXL1	16.66666667	0
CSF1R	16.66666667	0
DDR2	16.66666667	0
DIS3	16.66666667	0
EP300	16.66666667	0
FAM46C	16.66666667	0
FANCA	16.66666667	0
FANCI	16.66666667	0
FGF23	16.66666667	0
GALNT12	16.66666667	0
GEN1	16.66666667	0
HRAS	16.66666667	0
HSP90AA1	16.66666667	0
KDR	16.66666667	0

Gene	Group A (n=6)	Group C (n=7)
	Supersensitive	Insensitive
MEN1	16.66666667	0
MKNK1	16.66666667	0
MLL	16.66666667	0
MST1R	16.66666667	0
NFE2L2	16.66666667	0
NTRK3	16.66666667	0
PARP1	16.66666667	0
REL	16.66666667	0
RICTOR	16.66666667	0
RNF43	16.66666667	0
SOCS1	16.66666667	0
TNKS2	16.66666667	0
TSC2	16.66666667	0
VEGFA	16.66666667	0
ABL2	0	14.28571429
ACVR1B	0	14.28571429
AURKA	0	14.28571429
BCL2L2	0	14.28571429
CCNE1	0	14.28571429
CD79A	0	14.28571429
CDK12	0	14.28571429
CDK6	0	14.28571429
CDKN1A	0	14.28571429
CUL4A	0	14.28571429
EGFR	0	14.28571429
EPHA5	0	14.28571429
EPHA6	0	14.28571429
EPHB1	0	14.28571429
EPHB4	0	14.28571429
ERRFI1	0	14.28571429
FAM123B	0	14.28571429
FANCE	0	14.28571429
FANCL	0	14.28571429
FANCM	0	14.28571429
FGF10	0	14.28571429
FGF12	0	14.28571429
FGF14	0	14.28571429
FGF19	0	14.28571429
FGF3	0	14.28571429
FGF4	0	14.28571429
FGFR1	0	14.28571429
FH	0	14.28571429
GNA11	0	14.28571429
GNA13	0	14.28571429
GNAS	0	14.28571429

Gene	Group A (n=6)	Group C (n=7)
	Supersensitive	Insensitive
HSD3B1	0	14.28571429
IKBKE	0	14.28571429
IKZF1	0	14.28571429
IRS2	0	14.28571429
KDM5A	0	14.28571429
KEAP1	0	14.28571429
LTK	0	14.28571429
MEF2B	0	14.28571429
MITF	0	14.28571429
MRE11A	0	14.28571429
MYCN	0	14.28571429
NF1	0	14.28571429
NKX2-1	0	14.28571429
NOTCH1	0	14.28571429
NOTCH3	0	14.28571429
NSD1	0	14.28571429
PAK7	0	14.28571429
PARP2	0	14.28571429
PARP4	0	14.28571429
PIK3C2B	0	14.28571429
PIK3R2	0	14.28571429
PLCG2	0	14.28571429
PNRC1	0	14.28571429
POLE	0	14.28571429
RAD50	0	14.28571429
RAD51B	0	14.28571429
RANBP2	0	14.28571429
RBM10	0	14.28571429
STAT3	0	14.28571429
STK11	0	14.28571429
TEK	0	14.28571429
TOP2A	0	14.28571429
TP53BP1	0	14.28571429
TRRAP	0	14.28571429
WHSC1L1	0	14.28571429
XPO1	0	14.28571429
ZNRF3	0	14.28571429
ATR	16.66666667	28.57142857
PIK3CA	16.66666667	28.57142857
PTCH1	16.66666667	28.57142857
PTPRD	16.66666667	28.57142857
RET	16.66666667	28.57142857
TP53BP1	50	42.85714286
CDKN2B	33.33333333	28.57142857
